# Comparison of the Gut Microbiota Disturbance in Rat Models of Irritable Bowel Syndrome Induced by Maternal Separation and Multiple Early-Life Adversity

**DOI:** 10.3389/fcimb.2020.581974

**Published:** 2021-01-14

**Authors:** Wu Enqi, Song Jingzhu, Pei Lingpeng, Ling Yaqin

**Affiliations:** Key Laboratory of Ethnomedicine (Minzu University of China), Ministry of Education, Beijing, China

**Keywords:** microbiota, irritable bowel syndrome, rat, maternal separation, multiple early-life adversity, visceral hypersensitivity

## Abstract

**Background:**

The study aimed to identify the effects of modeling procedures on bacterial communities and to investigate whether different modeling procedures lead to consistent patterns of gut microbiome compositions.

**Methods:**

Two irritable bowel syndrome (IBS) rat models maternal separation (MS) alone and multiple-early-adversity modeling (MAM) were established and the gut microbiome were analyzed using 16S-rRNA-based high-throughput sequencing methods.

**Results:**

Rats from both models exhibited visceral hypersensitivity and the two model groups exhibited differences in the extent of visceral sensitivity and fecal water content. The microbial community structure of the two models exhibited significant differences compared to the controls, while the two model groups also exhibited significant differences between them. Furthermore, microbial community functional predictions suggested that the two models exhibited different abundances of metabolisms and pathways. Several common and distinct characteristic differences were also observed between the two model groups. *Alloprevotella* were more abundant in both model groups, while *Butyricicoccus*, *Turicibacter*, *Ruminococcus*, and *Clostridium_sensu_stricto* along with the family it belongs to were less abundant relative to controls. In addition, the abundance of *Clostridium_IV*, *Corynebacterium*, *Rothia*, *Elusimicrobium*, *Romboutsia*, *Allobaculum*, *Parasutterella*, and their related taxa were specifically associated with MS group, whereas *Butyricimonas* and *Vampirovibrio* along with its related taxa were specifically associated with MAM group. Among those, *Butyricimonas*, *Butyricicoccus* and *Corynebacterium* were found to partially mediate early adversity exposure-induced visceral hypersensitivity.

**Conclusions:**

Our results highlight the importance in evaluating gut microbiota characteristics in IBS research while also systematically considering potential modeling procedural differences. The microbial compositional/functional differences identified in this study were suggestive to further investigation of mechanisms of early adversity induced IBS.

## Introduction

Irritable bowel syndrome (IBS) is a chronic functional gastrointestinal disorder characterized by abdominal discomfort, bloating, and disturbed defecation in the absence of any identifiable abnormalities indicative of organic gastrointestinal disease (Holtmann et al., 2016). The pathophysiology of IBS is likely heterogeneous and may involve visceral hypersensitivity, gut barrier dysfunction, altered brain–gut signaling, immune dysregulation, altered microbiota characteristics, and psychosocial factors (Halland and Saito, 2015; Ford et al., 2017).

Various animal models have been developed to study the underlying mechanisms of IBS, among which the rodent maternal separation (MS) model is well-established and based on the premise that environmental changes during the early postnatal period can have long-lasting effects into adulthood, including visceral hyperalgesia and altered colonic motility (Fukui et al., 2018; Rincel and Darnaudery, 2019). Combined animal models have the advantage of allowing the mimicking of multiple etiological factors of IBS and are developed by exposing animals to multiple stimuli, as has been used in several IBS studies (Gong et al., 2014; Zhuang et al., 2016; Li et al., 2019).

Increasing data have demonstrated the relationship between alterations in gut microbiota characteristics and IBS. It was reported that IBS patients harbor altered gut microbiota compared to healthy controls, while fecal microbiota transplantation from healthy donors to IBS patients has shown promise in alleviating IBS symptoms (Tap et al., 2017; Aroniadis et al., 2019). Among animal model studies, rats exposed to early-life adversity, including maternal separation, intestinal inflammation, or infection, restraint stress, and water avoidance stress have been shown to display visceral hypersensitivity and altered microbiota profiles in adulthood (Ma et al., 2019; Rincel et al., 2019; Zhang et al., 2019). However, an understanding of the specific impacts of different modeling methodologies on gut microbiota is currently lacking, and the microbial signatures that are consistently associated with certain pathophysiological changes in IBS have not been identified.

In the present study, two common IBS rat models (MS alone and multiple early adversity modeling) were established. High-throughput microbial community analysis of their colon contents was then performed to identify the influence of modeling procedures on bacterial community and investigate whether different modeling procedures lead to consistent patterns of gut microbiota compositions. In addition, the possible mediating effects of gut microbial composition were explored between early adversity and altered pathophysiological variants.

## Materials and Methods

### Animals and Ethics Statement

Pregnant specific pathogen free (SPF) Sprague Dawley rats were obtained from SPF Biotechnology Co., Ltd. (Beijing, China), and arrived in the lab on day 15 of their pregnancies. Rats were housed in standard polypropylene shoebox cages (42 × 20.5 × 20 cm) on hardwood chip bedding in a designated room with controlled lighting (12:12 h light–dark cycle) at 22°C to 24°C and allowed free access to water and a standard diet. Pregnant rats were checked daily for litter, and the day of birth was defined as the postnatal day (PND) 0 for the litter. After delivery, the dams and litters were housed together until weaning (PND22). After weaning on PND22, male and female rats were separated, and three animals were kept in a single standard cage. Only male rats were included in this study to avoid the effects of hormones (n = 7 for each group). The experimental animal protocol was approved by the Ethics Committee of Minzu University of China (ECMUC2019005AO). The study was carried out in a Good Laboratory Practice (GLP) accredited laboratory.

### Study Design

Pregnant rats were randomly divided into three groups: the control (CTRL), MS, and multiple early adversity model (MAM) groups. Rats were left undisturbed in the CTRL group except for routine cage cleaning every 2 days. In the MS group, the maternal separation was performed once daily during PND1–PND21. Separations were conducted between 9:00 am and 12:00 noon by placing the offspring in plastic cages placed on the top of heating pads set at 30°C to 33°C in a separate room from the main holding room. In the MAM group, the maternal separation was performed once daily during PND1–PND21, acetic acid instillation was performed once daily during PND15–PND28, and the rats were restrained for 3 h every day during PND29–PND42. Rats are typically reared by PND43. Rats were anesthetized for acetic acid instillation, and 0.5% acetic acid was slowly instilled into the colon with a plastic tube (external diameter of 1 mm) at a depth of 6 cm from the anus and then maintained for 3 min. The acetic acid dose on the first day was 0.2 ml and was increased by 0.1 ml every day. After increasing to 0.5 ml, enemas were administered at a dosage of 0.5 ml every day. To model restraint stress, rats were placed in a transparent plastic restraint cylinder, in which they could move forward and backward but could not turn around for 3 h. All rats were weighed once a week after PND22. Abdominal withdrawal reflex (AWR) scores in response to colorectal distension were measured at PND78, and fecal water content was measured at PND82 followed by recovery of distal colon contents at PND84. All of the rats were anesthetized using Isoflurane and were sacrificed by exsanguinations after blood collected from the posterior vena cava on PND84 as described in previously (Parasuraman et al., 2010).

### Visceral Sensitivity Assessment

Visceral hypersensitivity was evaluated using the AWR score at PND78, as described previously (Xu et al., 2009; Wang et al., 2018). Briefly, a flexible balloon (6 cm) constructed from a surgical glove finger was attached to Tygon tubing and inserted 8 cm into the descending colon *via* the anus and held in place by taping the tubing to the tail. Rats were placed in transparent plastic cubicles (8 × 10 × 20 cm) and allowed to adapt for 10 min. colorectal distension was then established by rapidly inflating the balloon to constant pressure. Pressure was measured using a sphygmomanometer connected to a pressure transducer. The balloon was inflated to various pressures (20, 30, 40, 50, 60, 70, 80, 90, and 100 mm Hg) for a 10-s stimulation period followed by a 1-min rest period. Behavioral responses to colorectal distension were measured by visual AWR observations by two blinded observers, and AWR score assignments were as follows: 0, normal behavior without response; 1, slight head movement without abdominal muscle contraction; 2, contraction of abdominal muscles; 3, lifting of abdominal wall; and 4, body arching and lifting of pelvic structures. The minimal distention pressures that evoked abdominal visceromotor responses corresponding to AWR scores of 2, 3, and 4 were recorded by both observers and defined as the distension threshold.

### Fecal Water Content

Fresh feces expelled by each rat were collected on PND82 in 5 ml sterile tubes and immediately weighed, followed by drying them in an oven to calculate fecal water content with the following formula: water content% = [wet weight of the feces (g) − dried weight of the feces (g)]/wet weight of the feces (g) × 100%.

### Collection of the Colorectal Fecal Contents

Colorectal fecal contents were collected just after sacrifice. Fresh fecal pellets were collected directly from the distal colon, placed in 5-ml sterile tubes, and preserved with 4 M guanidine thiocyanate solution at −20°C, then transferred to the laboratory within 24 h and stored at −80°C prior to DNA extraction and characterization of the microbiota using 16S rRNA gene sequencing.

### Histological Examination of Colonic Inflammation

The histological tissues were fixed in formalin for 12 h, and pathological sections were generated at a thickness of 5 μm and processed for hematoxylin-eosin staining. The slides were observed under an optical microscope, and inflammatory cells were observed by a pathologist blinded to the treatments.

### Microbiota Sequencing

Total bacterial DNA was extracted from samples using the Power Soil DNA Isolation Kit (MO BIO Laboratories) according to the manufacturer’s protocol. DNA quality and quantity were assessed by the ratios of 260 nm/280 nm and 260 nm/230 nm. Then DNA was stored at −80°C until further processing.

The V3-V4 region of the bacterial 16S rRNA gene was amplified with the common primer pair (Forward primer, 5′-ACTCCTACGGGAGGCAGCA-3′; reverse primer, 5′- GGACTACHVGGGTWTCTAAT-3′) combined with adapter sequences and barcode sequences. PCR amplification was performed in a total volume of 50 μl, which contained 10 μl buffer, 0.2 μl Q5 High-Fidelity DNA Polymerase, 10 μl High GC Enhancer, 1 μl dNTP, 10 μM of each primer and 60 ng genome DNA. Thermal cycling conditions were as follows: an initial denaturation at 95°C for 5 min, followed by 15 cycles at 95°C for 1 min, 50°C for 1 min, and 72°C for 1 min, with a final extension at 72°C for 7 min. The PCR products from the first step PCR were purified through VAHTSTM DNA Clean Beads. A second round PCR was then performed in a 40-μl reaction which contained 20 μl 2×Phμsion HF MM, 8 μl ddH_2_O, 10 μM of each primer, and 10 μl PCR products from the first step. Thermal cycling conditions were as follows: an initial denaturation at 98°C for 30 s, followed by 10 cycles at 98°C for 10 s, 65°C for 30 s min, and 72°C for 30 s, with a final extension at 72°C for 5 min. Finally, all PCR products were quantified by Quant-iT™ dsDNA HS Reagent and pooled together. High-throughput sequencing analysis of bacterial rRNA genes was performed on the purified, pooled sample using the Illumina Hiseq 2500 platform (2×250 paired ends) at Biomarker Technologies Corporation, Beijing, China.

### Bioinformatic Analyses

Raw 16S rRNA gene sequence reads were demultiplexed and then trimmed, merged, and filtered using the Usearch v11.0.667linux32 program using the UNOISE pipeline. Paired-end sequence reads with an expected error (ER) value > 1.00 were removed during filtering. Sequences were dereplicated and classified into zero-radius operational taxonomic units (ZOTUs) after denoising (error-correcting) and chimera checking. The RDP classifier was then used to assign taxonomic classifications to 16S rRNA gene sequences with a confidence threshold of 80%. Representative sequences from the ZOTUs were aligned and further filtered to create a phylogenetic tree using the QIIME pipeline. The ZOTU table was subsampled randomly to the number of reads in the smallest sample (19,511) in order to obtain equal sequencing depths among samples. The functional profiles of the microbial communities were also predicted using the open-source R package Tax4Fun and the Kyoto Encyclopedia of Genes and Genomes (KEGG) ortholog dataset based on 16S rRNA gene profiles.

### Statistical Analyses

Categorical variables were evaluated as frequencies and percentages. Chi-squared tests and Fisher’s exact tests were then used to assess statistical associations between variables. Numerical variables were expressed as means ± standard deviations (SD). One-way analysis of variance (ANOVA) tests were used to compare differences in measured parameters among groups. Wilcoxon rank-sum test or Kruskal-Walls test were used to compare differences of nonparametric (not normally distributed) data

The Chao1, observed OTU numbers (obs_otus), phylogenetic diversity (PD_whole_tree), Shannon and Simpson indices were used to evaluate community richness and diversity of the gut microbial communities. Principal coordinates analysis (PCoA), adonis tests, and db-RDA analyses were used to evaluate differences among communities based on beta diversity metrics including the unweighted and weighted Unifrac distance metrics. Finally, the Linear discriminant analysis Effect Size (LEfSe) analysis with a threshold of +/−2 were used to explore significant differences among treatment groups using the relative abundance data of taxa and the KEGG-based ortholog and pathway abundances.

### Mediation Analyses

To further assess the contribution of alteration in gut microbiota to the association between early adversity and the altered physiological factors in adulthood including body weight, visceral hypersensitivity, and fecal water content, mediation analyses were conducted using the “mediation” package in R. Bacterial taxa were considered as mediators in mediation analysis to evaluate the apparent causal effects of early adversity on the physiological factors in adulthood. The total effect (TE) and average causal mediation effect (ACME) values were determined between predictors and outcomes *via* the mediators, and the percent of mediation effect was estimated using a two-tailed *P*-value cutoff of < 0.05 to assess statistical significance.

## Results

### Model Assessment

**Figure 1:**
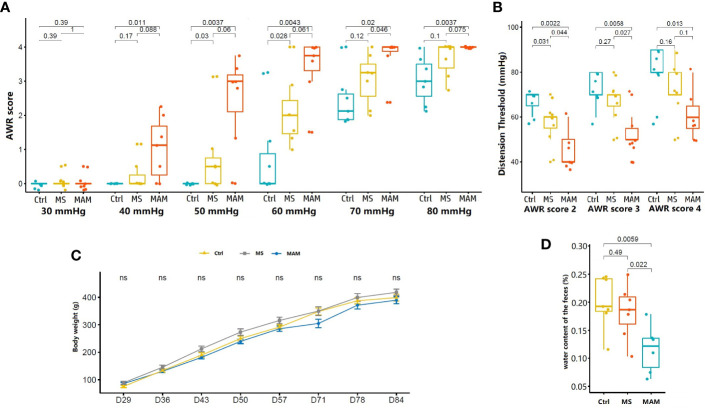
Comparison of visceral hypersensitivity, body weight, and fecal water content in rats of different model groups (n = 7 for each group). **(A)** Abdominal withdrawal reaction (AWR) scores in response to graded colorectal distension (CRD). **(B)** Distension threshold values for AWR scores of 2, 3, and 4. **(C)** Body weight variation of rats (ns, not significant). **(D)** Fecal water content for rats in the control, MS, and MAM groups.

Visceral sensitivity is one of the most important pathophysiological characteristics of IBS. Here, we used colonic stretch stimulation AWR scores to evaluate the visceral sensitivity of treatment groups. Both MS and MAM groups exhibited increases in visceral hypersensitivity to colorectal distension compared to control group individuals (**Figures 1A, B**). The MAM group showed even higher visceral hypersensitivity than did the MS group. The AWR scores were significantly higher in the MAM rats compared to those of the control group at pressures of 40, 50, 60, 70, and 80 mmHg and in MS rats at pressures of 50 and 60 mmHg. The threshold values for the MS group for AWR score 2 were significantly lower than those of the control group, while the threshold values of AWR scores 2, 3, and 4 were all significantly lower in the MAM group compared to the control group. These results thus indicate that visceral hypersensitivity was induced in both the MAM and MS groups.

No statistically significant differences were observed in body weights between different model groups, although the body weights of rats in the MS group were generally higher than those of the other two groups (**Figure 1C**). Fecal water content was measured to evaluate differences in general stool features, and a significant decrease in the fecal water content of MAM rats was observed compared to control rats (11.6% ± 4.3% vs 20.1% ± 4.7%, p = 0.0059, n = 7). No such effect was observed between the control and MS rats (18.3% ± 4.8%) (**Figure 1D**). Histological examination did not indicate remarkable inflammatory features in all groups on PND84, suggested that the two modeling methods did not cause obvious intestinal organic lesions.

### Overall Assessment of Intestinal Microbiota Variation

A total of 1,395,522 paired-end 16S rRNA gene sequence reads covering the V3–V4 hypervariable regions were generated from 21 samples, with an average of 66,453 (± 13,675.83 SD) reads per sample, and ranging from 45,296 to 80,200 per sample. After trimming and filtering, a total of 1,058,795 high-quality paired-end reads remained. Further denoising resulted in the removal of 1.091 chimeric sequences.

**Figure 2:**
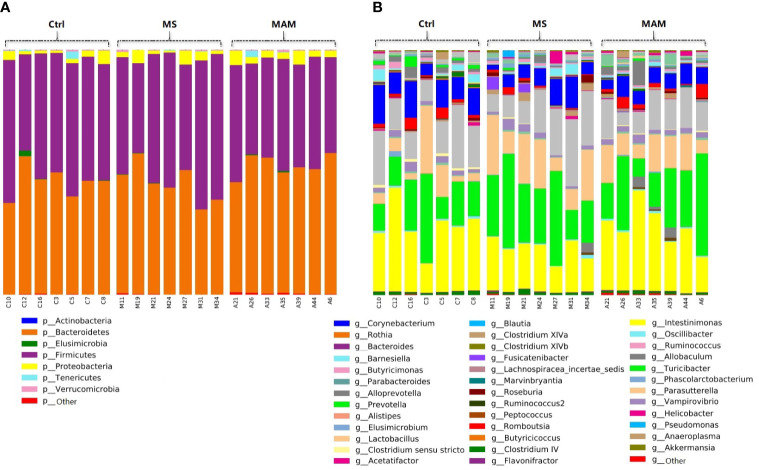
Bar plots of the gut microbiota compositions of each samples in 3 groups. **(A)** annotated at phylum level; **(B)** annotated at genus level.

A total of 4,498 zero-radius operational taxonomic units (ZOTUs) were ultimately generated and further used for data analysis. All ZOTUs were annotated by comparison against the RDP database. Among these, 4,453 ZOTUs were annotated at the phylum levels, comprising 7 phyla. In addition, a total of 4,401, 4,335, 4,125 and 1,940 ZOTUs were annotated at the class, order, family, and genus level comprising 13 classes, 14 orders, 21 families, and 38 genera, respectively (**Figure 2**).

### Comparison of Microbial Community Structures Among Different Model Groups

**Figure 3:**
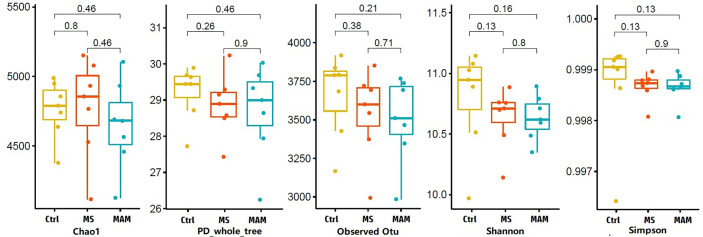
Alpha diversity values for gut microbial communities among different model group rats.

**Figure 4:**
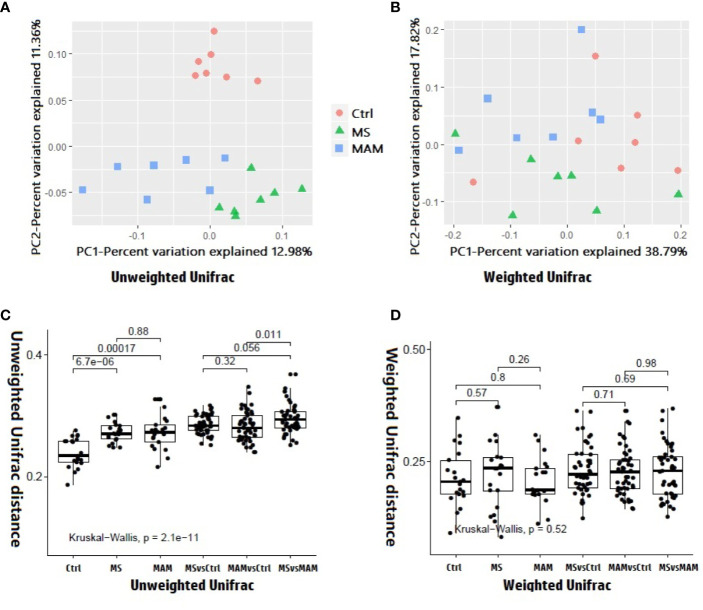
Beta diversity analysis among and within different model groups. **(A)** 2D PCoA plots showing differences in the gut microbiota of rats from different model groups based on the unweighted Unifrac distance metric. *p* = 0.001and r^2^ = 0.21127 in the Adonis test, *p* = 0.001 and adjusted r^2^ = 0.123634 in db-RDA analysis. **(B)** 2D PCoA plots showing differences in the gut microbiota of rats from different model groups based on the weighted Unifrac distance metric. *p* = 0.047 and r^2^ = 0.1743 in the Adonis test. *p* = 0.048 and adjusted r^2^ = 0.069004 in db-RDA analysis. **(C)** Comparison of inter-individual variation between and within different model groups based on the unweighted Unifrac distance metric. **(D)** Comparison of inter-individual variation between and within different model groups based on the weighted Unifrac distance metric.

Several different richness and diversity indices were calculated for the microbial communities of different treatment groups after randomly subsampling the ZOTU table to 18,429 reads per sample, which was the size of the smallest sample. No significant differences were observed for all five diversity indices among the different model groups (**Figure 3**). To examine bacterial community structural differences among model groups, two distance matrices were created from the subsampled ZOTU table based on the unweighted Unifrac distance metric and the weighted Unifrac distance metric. Principal coordinate analysis (PCoA) of beta-diversity differences based on the unweighted UniFrac distance metric indicated that the microbial communities from the three model groups were completely differentiated into three different clusters. Furthermore, PCoA based on the weighted Unifrac distance metric also indicated the partial separation of the microbiota communities based on the different model groups (**Figures 4A, B**). Adonis tests and db-RDA analyses of both distance matrices indicated the presence of significant associations between the different model groups and bacterial community structures.

Comparison of intra-group and inter-group distances for each model group based on the unweighted Unifrac distance metric suggested that the extent of variation within the MS and MAM groups was significantly higher than that of the control group, with no significant difference between the two model groups, indicating that the modeling process not only altered the structure of the gut microbiota, but the extent of instability of the gut microbiota also increased significantly. Pairwise comparisons of the distances between the groups indicated that the distance between the two model groups was significantly higher than the distances between either the MS or MAM communities to those of the control group, suggesting that the gut microbiota structure varies in different directions based on the modeling methodology (**Figure 4C**).

The comparison of the intra-group and inter-group distances of each model group based on the weighted Unifrac distance metric did not yield statistically significant differences (**Figure 4D**).

### Differences in Microbiota Composition and Functionalities Among Control and Maternal Separation Groups

**Figure 5:**
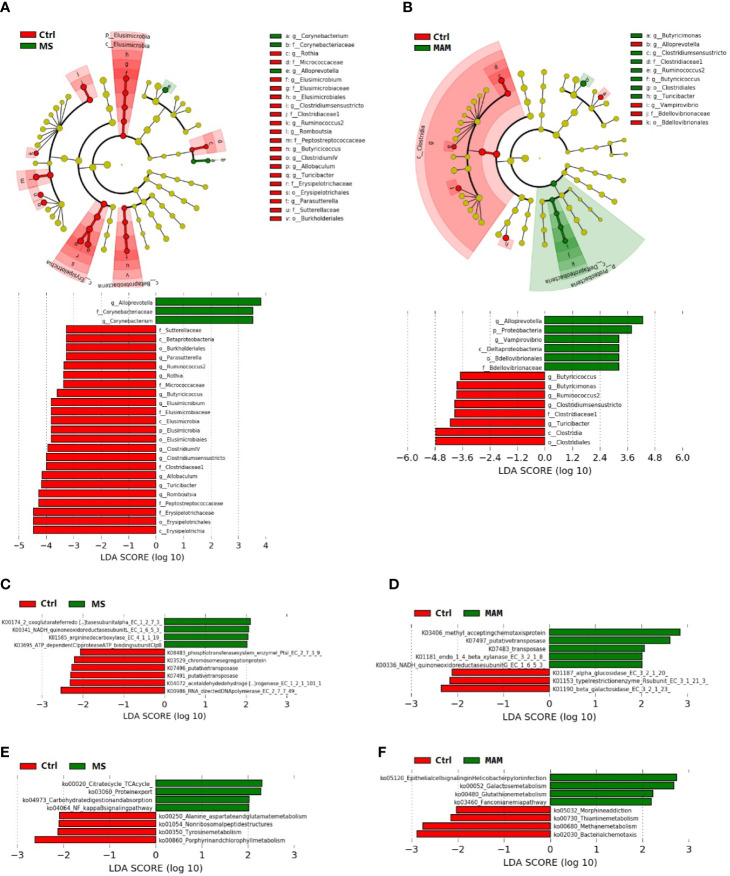
Linear discriminant effect size (LEfSe) analyses comparing differentially abundant taxa and the KEGG-based ortholog functions and pathways between control and MS group communities and control and MAM group communities. **(A, B)** The differences in gut microbiota taxa between different model groups. The phylogenic relationship of the taxon with differences were shown in the cladogram. **(C, D)** Differences of KEGG-based ortholog (KO) inferred functions in gut microbiota between different model groups. **(E, F)** Differences in KO pathways predicted from gut microbiota compositions between different model groups.

A total of 26 taxa exhibited significantly different abundances in the comparison between the control and MS groups (**Figure 5A**). *Corynebacterium* and the family to which it belongs as well as *Alloprevotella* were more abundant in the MS group. The genus *Rothia* and the family to which it belongs along with *Elusimicrobium* and the family, order, class, and phylum to which it belongs were more abundant in the control group. Other taxa that were more abundant in the control group communities included *Clostridium* sensu stricto and the family to which it belongs, *Ruminococcus*, *Romboutsia* and the family to which it belongs, *Butyricicoccus*, *Clostridium* IV, *Allobaculum* and the family, order, and class to which it belongs, *Turicibacter*, and lastly, *Parasutterella* and the family, order, and class to which it belongs.

In addition, 10 KOs and 8 pathways had significantly different inferred abundances between the control and MS groups (**Figures 5C, E**). Specifically, we observed higher abundances of the pathways in the MS group compared to the control group involving the citrate (TCA) cycle (ko00020), protein export (ko03060), carbohydrate digestion and absorption (ko04973), and NF-kappa B signaling (ko04064), along with lower abundances of alanine, aspartate, and glutamate metabolism (ko00250), non-ribosomal peptide structures (ko01054), tyrosine metabolism (ko00350), and porphyrin and chlorophyll metabolism (ko00860).

### Differences in Microbiota Composition and Functionalities Among Control and Multiple Early Adversity Model Groups

Comparison of the control and MAM groups indicated the presence of 14 taxa with significant differences (**Figure 5B**). Specifically, the genus *Vampirovibrio* and the family, order, class, and phylum to which it belongs as well as *Alloprevotella* were more abundant in the MAM group. In contrast, *Clostridium* sensu stricto and the family, order, and class to which it belongs along with the *Butyricimonas*, *Turicibacter*, *Ruminococcus*, and *Butyricicoccus* genera were more abundant in the control group samples.

LEfSe analysis indicated that 8 KOs exhibited significant differences comparing the control and MAM groups (**Figure 5D**). At the functional pathway level, 8 pathways had significantly different inferred abundances among the control and MAM groups (**Figure 5F**). In the MAM group, pathways that were significantly more abundant than in the control group included epithelial cell signaling in Helicobacter pylori infection (ko05120), galactose metabolism (ko00052), glutathione metabolism (ko004800), and the Fanconi anemia pathway (ko03460), while significantly lower pathways included morphine addiction (ko05032), thiamine metabolism (ko00730), methane metabolism (ko00680), and bacterial chemotaxis (ko02030).

### Mediation Analyses

Mediation analyses were performed to investigate the possibility that gut microbiota composition mediates the association between early adversity and altered physiological variants. Among the 26 taxa correlated with MS exposure, *Corynebacterium* variation was found to potentially mediate the association of MS exposure and the visceral hypersensitivity variable corresponding to the distention threshold score of 2 for AWR. Among the 14 taxa identified at different taxonomic levels described above that correlated with MAM exposure, variable abundances of *Butyricimonas* and *Butyricicoccus* were found to potentially mediate MAM exposure-induced abnormalities in visceral hypersensitivity (**Table 1**).

**Table 1:** Gut microbiota composition mediates the association between early adversity and altered physiological variants.

MAM exposure correlated alteration								
Taxa		Physiological Variants	ACME		Total		Prop. Mediated	
			beta	*p*	beta	*p*	%	*p*
*Butyricimonas*		AWR score at 50 mmHg	1.3	0.150	2.46	<0.001	52.44	0.030
*Butyricicoccus*		AWR score at 80 mmHg	0.5	0.170	0.97	<0.001	51.41	0.026
**MS exposure correlated alteration**								
Taxa		Physiological Variants	ACME		Total		Prop. Mediated	
			beta	*p*	beta	*p*	%	*p*
*Corynebacterium*		Distention Threshold for AWR score 2	−8.27	0.026	−10	0.018	80.79	0.044

ACME, average causal mediation effect; Prop. Mediated, proportion of mediation effect.

## Discussion

In the present study, we established two IBS rat models using maternal separation (MS) and multiple early adversities (MAM) modeling and investigated their intestinal microbiota. Rats from both IBS models exhibited visceral hypersensitivity compared with the control group rats. However, the two model groups exhibited differences in the extent of visceral pain sensitivity and fecal water content. The microbial community structure from rats with the two visceral hypersensitivity models exhibited significant differences compared to the controls, while communities from the two model groups also exhibited significant differences between them. Furthermore, functional predictions of the gut microbiota suggested that rats from the two models exhibited different abundances of metabolisms and pathways associated with microbiota structural changes. Several distinct and common characteristic differences were also observed between rats from the two model groups. Some of these bacterial taxa may play a mediating role in the association between early adversity and specific pathological changes of IBS.

Several studies have evaluated early adversity events and alterations in intestinal microbiota diversity. Some of these have suggested that exposure to early adversity can affect the gut microbiota, manifesting as a reduction in microbial diversity and richness (O’Mahony et al., 2009; Qian et al., 2019). However, other studies have reported no changes in diversity in these scenarios (Moya-Perez et al., 2017). Comparison of five alpha-diversity indices between different model groups did not yield any significant differences in this study. The apparent discrepancies in associations between microbial community alpha-diversity and early adversity could be explained by differences in study designs and environments, or even by factors of the same study including the type of early adverse events that were used and the timing of material collection, which can lead to variable results. For example, Zhou et al. (2016) evaluated the composition and diversity of the gut microbiota of MS, PI-IBS, and control rats at three different time points. No differences in alpha diversity were observed among the three groups in the third week and the eighth week, only the MS group exhibited a significant decrease in the Simpson and Shannon indices at week 12, while the PI-IBS group did not exhibit significant differences.

Inter-individual variation within host cohorts can also reflect the stability of gut microbiota. Healthy and homeostatic gut microbiota share higher similarities in the composition and richness of microbial taxa, while those with imbalanced microbiota exhibit drastic changes and tend to have higher inter-individual variation. Inter-group distances in the two model cohorts of this study exhibited significantly higher variation than those of the control group (**Figure 4C**). Thus, induced early negative events resulted in a significant increase in the instability of gut microbiota. exposure to early adversity can induce significant dysbiosis of fecal microbiota (Rincel et al., 2019).

To further evaluate the effects of different modeling processes on gut microbiota compositions, the composition and functionality differences of the communities among the model and control groups were further investigated. The two model groups exhibited several significantly altered taxonomic group abundances compared to the control group.

Bacterial taxonomic differences unique to the MS group involved 20 taxa related to 7 genera, namely, *Corynebacterium*, *Rothia*, *Elusimicrobium*, *Romboutsia*, *Clostridium* IV, *Allobaculum* and *Parasutterella*. Our finding that the abundances of *Parasutterella* species and their related taxonomic groups were significantly lower in the MS group compared to the control group was contrast with the result of Chen et al. (2018). The latter group found that *Parasutterella* was abundant in the stool of IBS patients. However, our results are consistent with other studies indicating that *Parasutterella* abundances are associated with beneficial outcomes. Zhang et al. (2019) reported that *Parasutterella* abundances were significantly lower in CDI patients and asymptomatic carriers than in healthy controls, while Kreutzer et al. (2017) also reported a negative correlation between *Parasutterella* abundances and high-fat diet-induced hypothalamic inflammation. Although the association of Parasutterella and different health outcomes is clearly controversial, these observations nevertheless indicate that *Parasutterella* has a role in modulating the microbial activities and host responses in certain disease states and, thus, necessitate further investigation. The associations identified here between *Corynebacterium*, *Rothia*, *Elusimicrobium*, *Romboutsia*, *Clostridium* IV, and *Allobaculum* with maternal separation stress have not been reported elsewhere. Consequently, their associations with IBS require additional confirmation in further studies.

The bacterial taxonomic differences unique to the MAM group comprised five taxa, where the genus *Butyricimonas* decreased significantly and *Vampirovibrio* along with the family, order, class, and the phylum to which it belongs all increased significantly compared to the control group. This results were consistent with the previous studies suggested an association and role for genera *Butyricimonas* and *Vampirovibrio* with intestinal function (Yang et al., 2017; Song et al., 2020). *Butyricimonas* have been reported to produce butyrate that reduces inflammation and helps maintain healthy gut functioning (Yang et al., 2017). *Vampirovibrio* has been reported as significantly more abundant in trinitrobenzene sulfonic acid-induced PI-IBS rat model individuals, and its lowered abundance has been associated with the alleviation of symptoms for visceral hypersensitivity after treatment (Song et al., 2020).

Predictive analysis of microbiota functionalities also suggested that changes in the microbial composition of the two model groups corresponded to functional differences associated with diverse metabolisms and pathways.

Specifically, we observed significant different abundances of the 8 pathways in the MS group compared to the control group. Among them, the enhanced functional capacity for the NF-kappa B signaling pathway in MS group is consistent with previous studies indicating that neonatal MS induces VH and visceral pain in rats that is mediated by activation of TLR4 and the NF-κB signaling pathway (Chen et al., 2015; Yuan et al., 2015) and further suggests that changes in the gut microbiomes caused by MS are involved in NF-κB activation in a rat model of IBS.

In the MAM group, we also found 8 significantly different pathways compared to the control group, which is distinct from that of MS group, suggest that different modeling strategies may result in IBS symptoms by inducing changes in microbiota *via* different metabolic pathways.

Despite the significant differences in microbial composition and functionalities among the two model groups, six taxa were common in both the model groups relative to the control group communities. Specifically, *Alloprevotella* were more abundant in both the MS and MAM groups, while *Butyricicoccus*, *Turicibacter*, *Ruminococcus*, and *Clostridium* sensu stricto along with the family it belongs to were less abundant in both the MS and MAM groups relative to controls. These results were consistent with those from previous studies. For example, *Alloprevotella* were significantly more abundant in model rats with early adversity stimuli compared to controls in one study (Rincel et al., 2019). In addition, *Turicibacter* were significantly less abundant in IBS-D patients (Zhuang et al., 2018). *Ruminococcus*, *Butyricicoccus*, and *Clostridium* sensu stricto are all short-chain fatty acid-producing genera that are considered beneficial for gastrointestinal tract functioning (Eeckhaut et al., 2013; Kong et al., 2019; Perez-Burillo et al., 2019). Furthermore, *Ruminococcus* spp. were less abundant in IBS patients relative to controls in another study (Mack et al., 2019). Moreover, several studies have shown that the abundances of *Butyricicoccus* were lower in ulcerative colitis patients and patients with inflammatory disease in general (Eeckhaut et al., 2013; Rios-Covian et al., 2016; Devriese et al., 2017). These microbial signatures exhibited similar abundance patterns in both early adversity modeling procedures, indicating that the responsive species may be involved in common characteristics of the complex pathophysiological changes that cause IBS. Thus, the variation in these taxa could provide clues to the etiology of IBS and potentially lead to novel therapies.

The changes in the gut microbiota and pathophysiological indicators described in this study were both induced by early adversity events. To further assess the contribution of altered gut microbial compositions to the association between early adversity and altered physiological outcomes, mediation analysis was performed to investigate the potential mediating effects of microbial populations on pathophysiological changes induced by early adversity events. *Corynebacterium* was predicted to partially mediate MS exposure-induced visceral hypersensitivity, while *Butyricimonas* and *Butyricicoccus* populations were predicted to partially mediate MAM exposure-induced visceral hypersensitivity. Thus, these genera might play critical roles in the development of early adversity-induced visceral hypersensitivity and may also represent targets for understanding the mechanisms underlying microbe-mediated visceral hypersensitivity.

A limitation of the present study is the lack of proven causal relationships between alterations in microbiota characteristics and visceral pain behaviors, despite the abundance of evidence suggesting that microbial compositions play roles in the development of visceral hypersensitivity. Future studies could deconvolute these effects by focusing on manipulating the gut microbiota through prebiotics, probiotics, or synbiotics to reverse the deleterious effects of early adversarial events on visceral pain or other IBS-related behaviors.

To conclude, the present study demonstrated that MS stress and multiple early adversity stress procedures could induce distinct alterations of the gut microbiota that also differ in stability, composition, and functionality in rat models. The large differences in gut microbiota between the two model groups coincide with the heterogeneity and complexity of IBS pathogenesis and highlight the importance in evaluating gut microbiota characteristics in IBS research while also systematically considering potential modeling procedural differences. The microbial compositional and functional differences identified in this study prompt further investigation of mechanisms of early adversity induced IBS related pathophysiological changes.

## Data Availability Statement

The datasets presented in this study can be found in online repositories. The names of the repository/repositories and accession number(s) can be found below: https://www.ncbi.nlm.nih.gov/, PRJNA609270.

## Ethics Statement

The animal study was reviewed and approved by the Ethics Committee of Minzu University of China.

## Author Contributions

WE, SJ, and LY performed the experiments. WE and SJ analyzed the data. PL and LY obtained funding for the project and planned the experiments. WE, PL, and LY prepared the manuscript draft for submission. All authors contributed to the article and approved the submitted version.

## Funding

The study of the rat’s physiological parameters and the histopathological analysis was funded by the foundation of Key Laboratory of Ethnomedicine (Minzu University of China), Ministry of Education (KLEM-ZZ201903, KLEM-ZZ2020GD01) and Master’s Independent Research Project of Minzu University of China (SSZZKY-20200027), the analysis of fecal microbiota was funded by the National Natural Science Foundation of China (81473451 and 81673769). The funding bodies had no role in the design of the study, collection, analysis, or interpretation of data or in writing the manuscript.

## Conflict of Interest

The authors declare that the research was conducted in the absence of any commercial or financial relationships that could be construed as a potential conflict of interest.

## Abbreviations

AWR, Abdominal withdrawal reflex; ACME, Average causal mediation effect; CTRL, Control; CRD, Colorectal distension; ER, Expected error; IBS, Irritable bowel syndrome; KEGG, Kyoto Encyclopedia of Genes and Genomes; KO, KEGG orthologs; LEfSe, Linear discriminant analysis Effect Size; MS, Maternal separation; MAM, Multiple early adversity model; ANOVA, One-way analysis of variance; PCoA, Principal coordinates analysis; PND, Postnatal day; SPF, specific pathogen free; SD, Standard deviations; TE, Total effect; ZOTUs, Zero-radius operational taxonomic units.
